# The conundrums of the reasonable patient standard in English medical law

**DOI:** 10.1186/s12910-023-00892-2

**Published:** 2023-02-23

**Authors:** Kelvin Hiu Fai Kwok, Eric C. Ip, Shing Fung Lee

**Affiliations:** 1 Faculty of Law, The University of Hong Kong, Pokfulam, Hong Kong SAR; 2 Asian Institute of International Financial Law, The University of Hong Kong, Pokfulam, Hong Kong SAR; 3 Centre for Medical Ethics and Law, The University of Hong Kong, Pokfulam, Hong Kong SAR; 4 Department of Radiation Oncology, National University Cancer Institute, National University Hospital, 1E Kent Ridge Road, NUHS Tower Block, Level 7, Singapore, 119228 Singapore; 5 Department of Clinical Oncology, Tuen Mun Hospital, New Territories West Cluster, Hospital Authority, 23 Tsing Chung Koon Road, Tuen Mun, Hong Kong SAR

**Keywords:** Reasonable patient standard, Informed consent, *Montgomery*, Medical negligence, Medical law, UK and Commonwealth

## Abstract

**Background:**

In its 2015 decision in *Montgomery v. Lanarkshire Health Board*, the Supreme Court of the United Kingdom overruled the long-standing, paternalistic prudent doctor standard of care in favour of a new reasonable patient standard which obligates doctors to make their patients aware of all material risks of the recommended treatment and of any reasonable alternative treatment. This landmark judgment has been of interest to the rest of the common law world. A judicial trend of invoking *Montgomery* to impose more stringent requirements on doctors is discernible in subsequent decisions since then.

**Main body:**

In this narrative review, without questioning the idea that properly informed patients should play a more active role in procedures affecting their own health in furtherance of their autonomy, safety, and consumer rights, we identify and analyse, with the aid of realistic clinical thought experiments, three practical conundrums that the *Montgomery* standard may inflict on the daily work of doctors, unfairly exposing them to arbitrary legal risks.

**Conclusions:**

These conundrums pertain to the ascertainment of the risks that must be disclosed to the patient under the test of ‘materiality’; the legal uncertainty as to the scope of the exceptions; and the actual ability of doctors to cope with the pressures of time. These conundrums offer ripe opportunities to rethink the proper role of judicially developed medical law in modern health care practice.

## Background

English medical law traditionally relies on what might be called a prudent doctor standard [1], as famously, or infamously, formulated in *Bolam v. Friern Hospital Management Committee* [2] which holds that doctors ought to follow ‘a practice accepted as proper by a responsible body of medical men’ in order to fulfil the standard of care expected of them in their diagnosis and treatment of patients. The House of Lords clarified in subsequent jurisprudence, as in *Sidaway v. Bethlem Royal Hospital Governors* [3], that doctors will not be negligent if they behave according to a practice accepted at the time by one group of responsible doctors, even if other doctors might reasonably follow a different practice. The prudent doctor standard is widely regarded as the last bastion of doctor-centric medical paternalism in English law [4]. The Supreme Court of the United Kingdom’s landmark decision in *Montgomery v. Lanarkshire Health Board* [5], delivered in March 2015, marked a significant legal shift [6], by propounding a ‘reasonable patient’ standard [7], which holds doctors liable to a duty to reasonably ensure that their patients are aware of any ‘material risk’ of recommended treatments, and of any reasonable alternative treatment [5]. Therefore, the law has turned away from inquiring what course of action a prudent doctor would have taken, and shifted to inquiring what sort of information a reasonable patient would have wanted to know from the doctor [8], which reflects a heightened emphasis on doctor-patient dialogues in contemporary health care.

## Main body

In this narrative review, without questioning the idea that properly informed patients should play a more active role in procedures affecting their health, in furtherance of their autonomy, safety, and consumer rights, we introduce the *Montgomery* standard and then identify and analyse—with the aid of hypothetical thought experiments which represent clinical encounters that may realistically happen and cannot simply be dismissed out of hand—three practical conundrums the *Montgomery* standard inflicts on the daily work of doctors, exposing them unfairly to arbitrary legal risks.

### The legal significance of the reasonable patient paradigm

The claimant in *Montgomery* was a woman who had given vaginal birth. Her baby was severely disabled due to complications in the delivery process. The plaintiff’s case was that the doctor in charge of her pregnancy and labour failed to properly advise her of the risk of shoulder dystocia in vaginal birth, and caesarean delivery as an alternative. The Supreme Court held that the doctor was indeed negligent under a new standard of informed consent. The risk of shoulder dystocia was a material risk that ought to have been disclosed, as the risk had been substantial (9–10%) that the baby would be seriously injured, and the doctor was aware of the plaintiff’s anxiety about her capacity to vaginally deliver the baby. The failure to disclose that risk constituted medical negligence on the facts. The Court offered several justifications for the new standard, *i.e.*:

- Firstly, patients are no longer passive recipients of medical services but consumers actively making choices.
- Secondly, patients have increased access in the information age to medical information outside of the doctor-patient relationship.
- Thirdly, ongoing developments in human rights law, medical practice, and the law of overseas jurisdictions support the new standard [5].

These factors together suggested that the law should move away from the medical paternalism presupposed by the *Bolam* standard, towards a new model in the UK which is founded upon patients’ informed consent and assumption of responsibility [5].

*Montgomery* has been hailed as affirming the status of informed patients as ‘masters of their own destiny,’ who may be presumed not to prefer that destiny to be ‘shaped unwittingly’ by their doctors [9]. In fact, variants of this new standard already existed in Australia, Canada, and the United States [10–12]. Indeed, the Supreme Court relied heavily on the Australian High Court’s decision in *Rogers v. Whitaker* [10]. Given the transnational influence of the British Court, it was anticipated that *Montgomery* would sooner or later be adopted by the courts of dozens of other Commonwealth jurisdictions that still adhered to the old *Bolam* standard. Singapore, for example, has followed suit in 2017 [13]. Notwithstanding the many benefits of such a flexible standard in advancing patients’ autonomy, safety, and consumer rights [5], *Montgomery* has become the target of much criticism in the bioethics literature [14]. The ruling has been faulted by two notable commentators, for instance, for ‘infantilising the patient’ and ‘demonising the doctor’, and its standard for being internally contradictory, for a doctor’s decision whether to disclose material risks to the patient has been treated as a non-medical judgment, whereas his assessment of whether a disclosure would undermine a patient’s health is being considered a medical one [15].

### Practical conundrums of the reasonable patient standard

In the following, we put forward the view that the standard formulated in *Montgomery*, obligating doctors to customise their approaches to the needs and conditions of each patient any advice they give about risk, is likely to pose significant challenges to the daily work of medical practitioners in the UK and in other countries apt to follow British jurisprudence. We highlight below three major practical conundrums, contextualised in clinical cases. The first conundrum pertains to the ascertainment of the risks that must be disclosed to the patient under the test of ‘materiality.’ While it seems intuitively right that doctors ought to disclose all the material risks of a medical procedure when obtaining patients’ informed consent, ascertaining which risks are material has been a challenge. The Supreme Court in *Montgomery* propounded the following test of ‘materiality’: a risk is material if within the particular case: (1) a reasonable person in the patient’s shoes would likely attach significance to the concerned risk; or (2) the doctor is or reasonably ought to be aware that the patient would likely attach to it significance [5]. The first aspect of the test defines materiality from the standpoint of a ‘reasonable person in the patient’s position’; the second defines it from the standpoint of the particular patient’s likely concerns, while constructing the doctor’s awareness of that. In considering what a reasonable person in the patient’s shoes would think, it is essential to be sensitive to the patient’s characteristics when it comes to giving advice on material risks. The problem, however, is that not all patient characteristics come within a doctor’s bounded attention-span, and to become fully aware of them during the doctor’s time-limited contact with the patient is humanly impossible. And it is not just medical characteristics that the legal rule implies; each and every characteristic of a patient, psychological, social or otherwise, might entail an individual material risk. Consider the following example:

Patient 1 is a male in his 70’s. He divorced a few years ago, with no children. He has recently started a new relationship with a 35-year-old woman. He very much wants to have children with her, but he has not disclosed this desire to his doctors to avoid embarrassment. He has been suffering from bilateral inguinal hernia with an increase in symptoms lately. He has thus been referred to a surgeon for repair operation. The operation entails the risk of infertility due to azoospermia, which, despite being a small risk (< 0.01%), is important to Patient 1 given his overwhelming desire to have children. The surgeon, considering Patient 1’s old age, and not knowing his desire, decides not to mention the risk before the operation. The risk unfortunately materialises and Patient 1 is not able to father children for the rest of his life.

What triggers the materiality of the risk of infertility in this example is the patient’s desire to have children despite his old age. Under the *Montgomery* test, it arguably qualifies as a material risk in the first category, because a reasonable person in the patient’s shoes, given *this* patient’s particular desire, would likely attach significance to such a very small risk. But it would seem very unfair to hold the surgeon liable for failing to properly disclose the risk, as what triggered its materiality—the desire to have children—was something the doctor was unaware and could not reasonably have become aware of. The non-applicability of the second category of material risks notwithstanding, the doctor remains fully liable for failing to disclosure a first category risk.

The second conundrum lies in the scope of the permitted exceptions, a topic that the Court in *Montgomery* explicitly declined to give any detailed guidance [5]. The Court merely identified exceptions to the general requirement of material risk disclosure [5]. The first is waiver; *i.e.*, the patient has made it clear that ‘she would prefer not to discuss the matter’. The second is the ‘therapeutic exception’; *i.e.*, risk disclosure is not required if the doctor ‘reasonably considers that its disclosure would be seriously detrimental to the patient’s health’. There may be instances where this exception clearly is pertinent, as when the doctor is reasonably certain that the patient would react to the risk disclosure by attempting suicide. Many other cases are not nearly so clear-cut, such as the following one, inspired by the facts of an actual but rare case from Australia in which the therapeutic exception was thought to be applicable [16, 17]:

Patient 2 is a man who suffers from severe depression with a strong tendency to attempt suicide. His doctor is considering treating his mental illness with high dosages of an antipsychotic drug that comes with the potential side-effect of serious eye damage. The doctor is reluctant to disclose this risk given the possibility that disclosure would aggravate the side-effect by causing the Patient 2 to react emotionally and suffer from immediate blindness. The doctor decides to withhold disclosure and proceed with the treatment, but this unfortunately results in permanent eye damage on the part of the patient, who now sues the doctor for medical negligence.

It may be argued that respect for the patient’s autonomy would require disclosure in order for the patient to make an informed choice over whether or not to accept treatment, albeit a choice that may not serve the patient’s best interests from the doctor’s perspective [5]. Indeed, the patient’s autonomy should normally be respected so long as he has the necessary decision-making capacity to provide consent to treatment, notwithstanding his disordered mental state. The doctor’s concern, however, is that pre-treatment disclosure may possibly result in immediate, grave harm to the patient. But whether the harm would materialise in the course of treatment still depends on the extent of his reaction to the disclosure and the effect of any overreaction on the development of side-effects. It may not be easy for the doctor to predict the patient’s reaction or the relevant health implications in a given situation. Much depends on the patient’s emotional state and his health concerns. In cases like the example above, a therapeutic exception unfairly burdens doctors with difficult choices.

The third exception identified by the Court to the general rule of material risk disclosure is necessity or emergency; *i.e.*, disclosure is not necessary where the patient needs urgent treatment but is unable to make any decision. However, what counts as an ‘emergency’ is not always clear. Consider this scenario:

Patient 3 is undergoing an appendectomy for acute appendicitis. During the operation, the surgeon discovers a malignant-looking mass in another part of the large intestine. This mass is found to be locally advanced with invasion to surrounding structures, but is still resectable. Although informed consent was obtained from Patient 3 before the operation, she only gave consent for the appendectomy. It is impossible to reverse the anaesthesia at this moment to explain to the patient the risks concerning the excision of an incidental lesion in the same operation.

It is unclear in this example whether removing the lesion is ‘urgent’ enough to justify immediate medical intervention without prior discussion of risks. Postponing treatment would allow a better assessment and pre-operative preparation, but the delay might in theory adversely affect the prognosis. A surgeon would be on the fence about whether to proceed with lesion removal in the same operation or postpone it until informed consent can be obtained. Lack of clarity on the scope and applicability of the therapeutic and necessity or emergency exceptions will likely impose an extra layer of legal uncertainty on doctors faced with hard medical situations. One should bear in mind that doctors can be held negligent not just for unreasonably relying on one of the exceptions in failing to disclose risks, but also for unreasonably relying on the informed consent approach in failing to proceed speedily to treatment; *i.e.*, *not* invoking an exception to avoid disclosure when it should be avoided.

The third conundrum relates to time pressure. Indeed, the Court noted this problem: it is not possible to discuss the risks of a medical procedure inside the time available for a typical health care consultation, but offered no solution [5]. A doctor faced with a patient who is nervous or obsessive about risk would naturally be expected to slow down, answer more questions, perhaps advise on a wider range of risks and of alternative procedures. This higher expectation may be consistent with the patient-sensitivity of the informed consent standard [5], but it may fail to accommodate the time pressure and resource constraints under which many doctors and hospitals operate. Consider the following:

Patient 4 suffers from potentially curative lung cancer and is being counselled on pneumonectomy. He accepts all relevant surgical and anaesthetic risks following his doctor’s detailed advice. However, he is fixated on the small risk of the procedure leaving a permanent needle mark on his arm following blood taking, and presses his doctor for details of the blood taking procedure and the needle mark. The doctor is facing increasing time pressure with a long queue of patients waiting for consultation.

In this thought experiment it is questionable whether the doctor would have much time to spend on a minor side effect of the treatment. In reality, a doctor dealing with a nervous or obsessive patient in a similar situation would have to stop at some point despite running the risk of legal liability for inadequate disclosure. Spending more time with the patient may in fact save time and sources down the line when disputes over side-effects arise. In a resource-constrained work environment, however, the doctor may not have much control over the consultation time for each patient. In these circumstances, the strict application of the *Montgomery* standard may result in unnecessary and arbitrary legal liability on Patient 4’s doctors.

## Conclusions

Our point in this narrative review is not that it is wrong for patients to take a more active part in making prudent choices for themselves or in responding to their doctors’ reasoned explanations and advice [18]. We are of the view, however, that the reasonable patient standard erected by *Montgomery*, applied in an unnuanced form, is not well-attuned to the realities of medical practice, and at times may be impossible to comply with [19]. The *Montgomery* standard was developed by neither doctors nor patients, but judges, the vast majority of whom lack medical qualifications or extensive health care experience, in their capacities as third-party dispute-resolvers. The three conundrums identified above offer rich opportunities for us to rethink whether judges are really best positioned to pass judgment on the niceties of medical procedure [20]. There is evidence suggesting that the judicial trend in post-*Montgomery* UK is to interpret the reasonable patient standard expansively to cover post-operative and pre-operative procedures, and to impose more stringent requirements on the timing of doctors’ disclosures to patients about risks [21]. But it appears that some judges elsewhere in the Commonwealth are beginning to realise the limits of the material risk disclosure requirement. In *Wallace v. Kam* [22], decided two years before but not cited in *Montgomery*, the Australian High Court held that doctors are not negligent even if they did not disclose a material risk, insofar as it can be shown, as in the instant case, that the patient would anyway not have acted differently had it been disclosed [23]. In the UK there have been unsystematic and uneven attempts to water down *Montgomery* case after case [21]. It remains to be seen whether the English version of the reasonable patient standard would give due recognition to real-world constraints on medical practice [24] without, of course, excusing preventable harm merely in the name of resource constraints and the like.

## Data Availability

Data sharing is not applicable to this article as no datasets were generated or analysed during the current study.
